# Evaluation of Immune Infiltration Based on Image Plus Helps Predict the Prognosis of Stage III Gastric Cancer Patients with Significantly Different Outcomes in Northeastern China

**DOI:** 10.1155/2022/2893336

**Published:** 2022-03-25

**Authors:** Tianyi Fang, Zhidong Wang, Xin Yin, Hao Wang, Lei Zhang, Xuan Lin, Xinghai Zhang, Yimin Wang, Yingwei Xue

**Affiliations:** ^1^Department of Gastrointestinal Surgery, Harbin Medical University Cancer Hospital, Harbin Medical University, Harbin 150081, China; ^2^Department of Hepatopancreatobiliary Surgery, Second Affiliated Hospital of Harbin Medical University, Harbin Medical University, Harbin 150081, China; ^3^Department of Pathology, Harbin Medical University, Harbin 150081, China; ^4^Department of Gastrointestinal Surgery, The First Affiliated Hospital of Zhejiang Chinese Medical University, Hangzhou 310006, China

## Abstract

Gastric cancer (GC) might have significantly different outcomes within the same AJCC/UICC-TNM stage. The purpose of this study is to help predict the different prognosis through the pattern of immune cell infiltration. We retrospectively analyzed 2605 patients who underwent radical gastrectomy in the Harbin Medical University Cancer Hospital between 2002 and 2013. For stage III with significantly different survival probability, we analyzed the relationship between immune cell surface antigen and survival in TCGA dataset. Furthermore, 200 cases in stage III GC with different survival outcomes were randomly selected for immunohistochemical verification. Image Plus software was used to evaluate the area of immune cell infiltration. We found that patients in stage III had significantly different outcomes. Bioinformatics analysis showed that there was a significant negative correlation between the expression of immune cell surface antigen and prognosis. In order to investigate whether immune infiltration can distinguish GC patients in stage III with differences in prognosis, we verified by immunohistochemistry that CD4^+^ T cells, CD20^+^ B cells, and CD177^+^ neutrophils infiltrated more in group B with better prognosis; CD8^+^ T cells, CD68^+^ macrophages, and CD117^+^ mast cells infiltrated more in group A with poor prognosis. CD117^+^ mast cells have the same trend of predicting significance for prognosis in the RNA and protein levels. In conclusion, patients with GC in northeastern China have significant prognostic differences only in stage III. CD117^+^ mast cells may be important evaluation factors in further studies of Immunoscore.

## 1. Introduction

Gastric cancer (GC) is one of the most common malignancies in the world [1]. Since 1968, the traditional AJCC/UICC-TNM classification (tumor burden, lymph nodes, and evidence of metastases) has been widely accepted as a standard of predicting the prognosis and even as a guide for further treatment for cancer [2–4]. However, with the progress of research on molecular medicine, patients needed more prognostic information about predicting the response to chemotherapy [5, 6]. In 2010, the Gastric Cancer Working Group first reported that there might be significantly variable outcomes in GC patients within the same TNM stage because of different immunotherapy [7].

This is due to the fact that the traditional TNM staging system relies entirely on the progression of tumor cells and fails to consider and incorporate the effects of immune responses [8], whereas in pathological sections of GC tissues, many types of immune cells can be identified, such as neutrophils, macrophages, mast cells, T cells, B cells, and NK cells, with their own characteristic infiltration patterns rather than being randomly distributed [9–12]. Pathological immune assessment thus may provide more insights on the prognosis of GC [13–15]. Therefore, a comprehensive assessment of these immune cells is critical. In 2012, Galon et al. [16] first proposed the definition of TNM-I (TNM-Immune), which is also called Immunoscore. In order to define the outcomes of cancer patients better and improve the quality of life by predicting patients who will benefit from adjuvant therapies, they initiated an international task force to incorporate the Immunoscore as a new component for the classification of cancer.

Immunoscore seem to be an ideal method [8, 17–20]. However, the limitations that the tumor-infiltrating immune cells appeared heterogeneously in the center of the tumor, the invasive margin of tumoral nests or the adjacent lymphoid structures, have made it difficult for researchers to agree on the selection of evaluation area [16]. Therefore, it is difficult to obtain stable and widely recognized results. Another technical barrier in studying tumor immunity lies in the inherent complexity of immunohistochemistry, in which the relationship between immune cells and cancer cells is extremely difficult to describe. The established models based on in vitro cell lines cannot truly reflect the intricate tumor microenvironment [21]. These pitfalls warrant the need to study immune cell infiltration from gene expression level to show whether it is the different forms of infiltrated immune cells that reflects the unique potential biology of tumors [22, 23]. The analysis of transcriptome sequencing data from real specimens can provide a more comprehensive background and help us unveil the real relationship between immune cells and GC. In addition, changing the validation cohort from consecutive patients to patients with extremely different outcomes may reduce false positive outcomes.

In this study, we first retrospectively analyzed the consistency between the prognosis of GC patients and traditional AJCC/UICC-TNM classification. We found that the prognosis of patients with stage III was significantly different, which was difficult to evaluate uniformly. Based on the transcriptome sequencing data of TCGA database, this study analyzed the relationship between the expression of common immune cell surface-specific antigen and the clinical clinicopathological features and calculated the relationship between mRNA expression and prognosis in stage III GC. Considering the clinical practicability, we used immunohistochemical method to evaluate the difference of immune cell infiltration in patients with significantly different prognosis of stage III GC. The expression level of immune cells was evaluated by Image Plus software calculating the percentage within the unit field of view. Immunological indicators based on traditional pathology and transcriptomics can complement the TNM 8th edition, which had significance for predicting outcomes of GC patients.

## 2. Materials and Methods

### 2.1. Patients and Tissue Specimens

We retrospective analyzed GC patients between 2002 and 2013 admitted into the Department of Gastrointestinal Surgery of the Harbin Medical University Cancer Hospital in this study. All patients had no tumor-invading surrounding tissues. The diagnosis was based on paraffin sections obtained preoperatively by electronic fiberoptic gastroscope and/or confirmed postoperatively by an experienced pathologist. All patients were performed hematology, abdominal ultrasound, electrocardiogram, gastric computed tomography (CT)/magnetic resonance imaging (MRI), chest radiograph, and abdominal ultrasound, and some patients were performed positron emission tomography (PET) when necessary. Our research center is a Gastric Cancer Diagnosis and Treatment Center of Heilongjiang Province, in Northeast China, and all operations were performed by the chief physician. In order to control the quality of the operation, photos and tables were recorded for each operation. The inclusion criteria were as follows: all patients with primary GC underwent radical gastrectomy and D2 lymph node dissection, availability of follow-up data and clinicopathological characteristics (median follow-up time was 47 months), and no history of chemotherapy before operation. All patients signed an informed consent.

We randomly selected 200 patients with significantly different outcomes (survival less than 1 year or more than 5 years) in stage III GC as the validation set, with the same inclusion criteria as above, from the patients' datasets for comparison purpose. Briefly, 100 patients with survival time less than 1 year were randomly selected as group A and another 100 patients whose survival time was more than 5 years as group B. This study was approved by the Ethics Committee of the Harbin Medical University Cancer Hospital (Ethical Approval number: 2019-57-IIT), and all procedures were carried out in accordance with ethical principles. All clinical information was retrieved from the Gastric Cancer Information Management System v1.2 of the Harbin Medical University Cancer Hospital (Copyright No. 2013SR087424, http://www.sgihmu.com/) including sex, age, Borrmann type, pTNM stage, and serum tumor marker test. The pTNM stage was according to the 8th edition American Joint Committee on Cancer (AJCC). All patients were reexamined by ultrasound, CT and gastroscopy, and tumor markers at least once a year, and PET/CT was performed as needed.

### 2.2. Bioinformatics Data Analysis

Gene expression profiling datasets were obtained from The Cancer Genome Atlas (TCGA) (https://tcga-data.nci.nih.gov/tcga/) including the mRNA sequencing of 407 samples (375 tumor tissue samples and 32 adjacent nontumor tissue samples) in all stages and 149 samples in stage III. R2 Genomics Analysis and Visualization Platform (http://r2.amc.nl) was used to analyze the relationship between mRNA expression of CD4, CD8, CD20, CD56, CD68, CD117, and CD177 and T, N, and M stages. The mRNA data of patients in TCGA database were selected and the prognostic correlation was performed by R studio.

### 2.3. Immunohistochemistry

Paraffin sections from the 200 cases of GC patients were dewaxed in xylene and ethanol. After cleaned in distilled water, the paraffin sections were pretreated with citrate buffer, pH 6.0 (CD177) and EDTA Antigen Retrieval Solution, pH 8.0 (CD4, CD8, CD20, CD56, CD68, and CD117) for 3 min at 120°C in a pressure cooker, and endogenous peroxidase was inhibited with 3% H_2_O_2_ in PBS for 10 min. Nonspecific actions in the sections were also blocked with goat serum (BOSTER, USA) for 1 h at room temperature. The sections were then incubated with the primary antibody overnight at 4°C, followed by incubation with the secondary antibody for 30 min at 37°C. Primary antibodies used were CD4 (ab183685, 1 : 1000, Abcam, Cambridge, MA, USA), CD8 (ab4055, 1 : 100, Abcam, Cambridge, MA, USA), CD20 (ab9475, 1 : 50, Abcam, Cambridge, MA, USA), CD56 (ab75813, 1 : 100, Abcam, Cambridge, MA, USA), CD68 (ab213363, 1 : 4000, Abcam, Cambridge, MA, USA), CD117 (ab32363, 1 : 400, Abcam, Cambridge, MA, USA), and CD177 (ab220279, 1 : 200, Abcam, Cambridge, MA, USA). Second antibodies used were goat anti-rabbit IgG (CD4, CD8, CD56, CD68, CD117, and CD177) and goat anti-mouse IgG (CD20). The chromogenic reaction was performed via diaminobenzidine (DAB) staining, and the staining intensity was measured using Image-Pro Plus version 6.2 software (Media Cybernetics, Rockville, Maryland, USA).

### 2.4. Evaluation of Immunohistochemical Staining

All specimens were examined blindly by two independent pathologists based on the staining percentage of positive cells. In order to eliminate the heterogeneity of immune cell distribution to the greatest extent, a series of optimal experimental processes was carried out to reduce the deviation. Pathologists without knowing the identity of the patients carefully examined the H&E staining of multiple wax blocks from the same patient sample before the experiment. The most representative blocks, which covered multiple heterogeneous regions, were selected to prepare tissue sections for experiment with the same criteria [24, 25]. To minimize the impact of spatial heterogeneity, the image information was collected from lymphocyte enrichment area, interstitial area, and tumor cell enrichment area, respectively, and the average area of results was estimated as relative percentage staining and intensity staining. Images of three representative fields at ×200 magnification were captured, and the areas of immunostaining in each image were measured using Image-Pro Plus version 6.2 software. The results were quantified as immune marker positive area/total area. In addition, all the selected patients were without preoperative chemotherapy and preoperative radiotherapy to eliminate the effects of chemotherapy drugs and radiation on tumor cells and immune cells.

### 2.5. Statistical Analysis

Data were presented as the mean ± standard deviation (SD) of this research and processed using SPSS 22.0 software (SPSS, Inc., Chicago, Illinois, USA). *T*-test was applied to analyze the significance of difference between groups. The rank sum test was used to analyze the significance of difference between the immunohistochemical positive area from groups A and B. The overall survival (OS) was calculated from the date of surgery to the last follow-up or date of death from any cause. Survival analysis was tested using the Kaplan-Meier method with log-rank test. The correlation between variables was tested by calculating Pearson's correlation coefficient. A two-tailed *P* < 0.05 was considered statistically significant.

## 3. Results

### 3.1. Patient Characteristics

**Figure 1:**
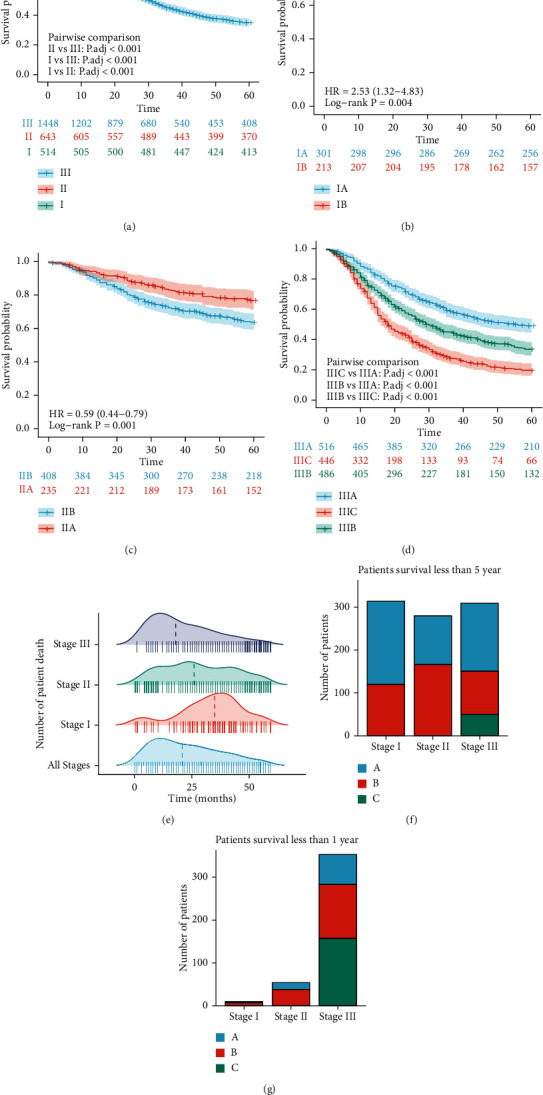
The relationship between the OS probability and the TNM stage. Kaplan-Meier survival analysis of OS probability according to the 8^th^ TNM stage of patients with GC (a), in stage I (b), in stage II (c), and in stage III (d). (e) Number of GC patients died in each month during 5 years. (f) Number of patients with different TNM stage GC with a survival time of more than 5 years. (g) Number of patients with different TNM stage GC with a survival time less than 1 year.

From 2002 to 2013, 2605 patients including 1934 males and 671 females received radical gastrectomy in our department and received conventional chemotherapy after surgery according to the physician's assessment. The overall 5-year survival rate was 45.7% (survival curves of patients in each stage are shown in Figures 1(a), 1(b), 1(c), and 1(d)). The numbers of patients in each pathological tumor-node-metastasis (pTNM) stage were 301 in IA, 213 in IB, 235 in IIA, 408 in IIB, 516 in IIIA, 486 in IIIB, and 446 in IIIC.  Figure 1(e) shows the number of deaths in different survival time of patients within 5 years. We found that the number of deaths in patients reached maximum at 8-12 months postsurgery and decreased gradually afterwards.  Figure 1(f) shows the distribution of pTNM stages in patients who survived more than 5 years including 408 patients in stage III.  Figure 1(g) shows the distribution of pTNM stages in patients died less than 1 year postsurgery including 353 patients in stage III, accounting for 84.7%. In the patients survived more than 5 years, although the proportion in stage III decreased to 34.3%, the total number did not decrease significantly. Therefore, there is a difference in the survival of patients with stage III GC after radical resection, and it is obvious that the existing TNM staging system has a limited value to predict the 1-5-year prognosis of patients with stage III GC.

In order to evaluate the prognostic effect of different immune cells more accurately, we selected two groups with completely different prognostic outcomes from stage III GC. Immunohistochemistry was used to evaluate the infiltration of immune cells in group A (survival time less than 1 year) and group B (survival time more than 5 years). The clinical characteristics of patients in groups A and B are shown in Table 1.

### 3.2. Transcriptome Analysis of GC Patients Based on TCGA Datasets

**Figure 2:**
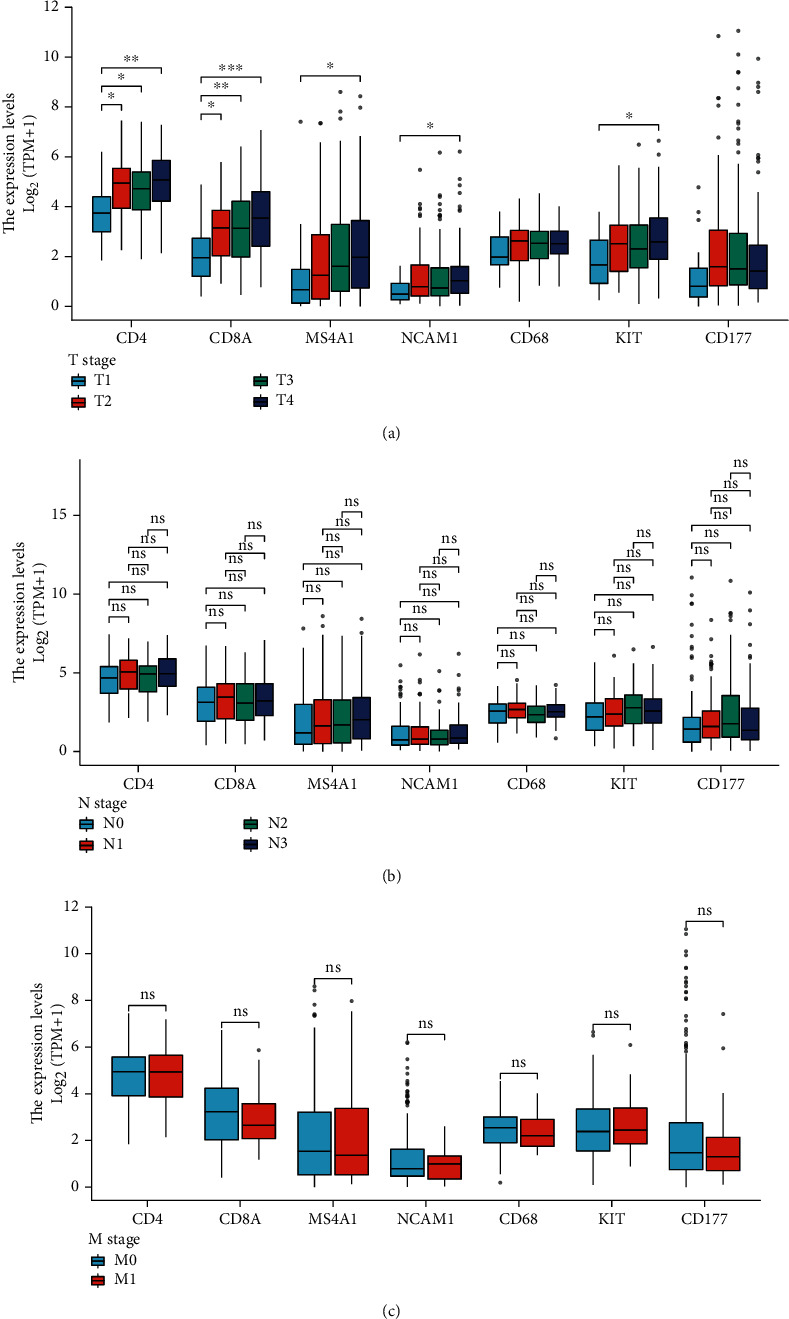
The relationship between expression of immune cell surface antigen in transcriptome level and T (a), N (b), and M (c) stage of GC patients. ^∗^*P* < 0.05, ^∗∗^*P* < 0.01, and ^∗∗∗^*P* < 0.001.

Immune cells might be closely related to the progress of GC. To verify this hypothesis, we divided the patients into different groups according to T, N, and M stages: T1, T2, T3, and T4 (Figure 2(a)) and N0, N1, N2, and N3 (Figure 2(b)) and M0 and M1 (Figure 2(c)). We found that with the increase of T stage, CD4, CD8, CD20, CD56, and CD117 mRNA expressions were increased. But there was no significant difference in expression between groups at different N or M stages, which indicated that the local effect of immune cells had a greater influence on local infiltration of tumor cells.

**Figure 3:**
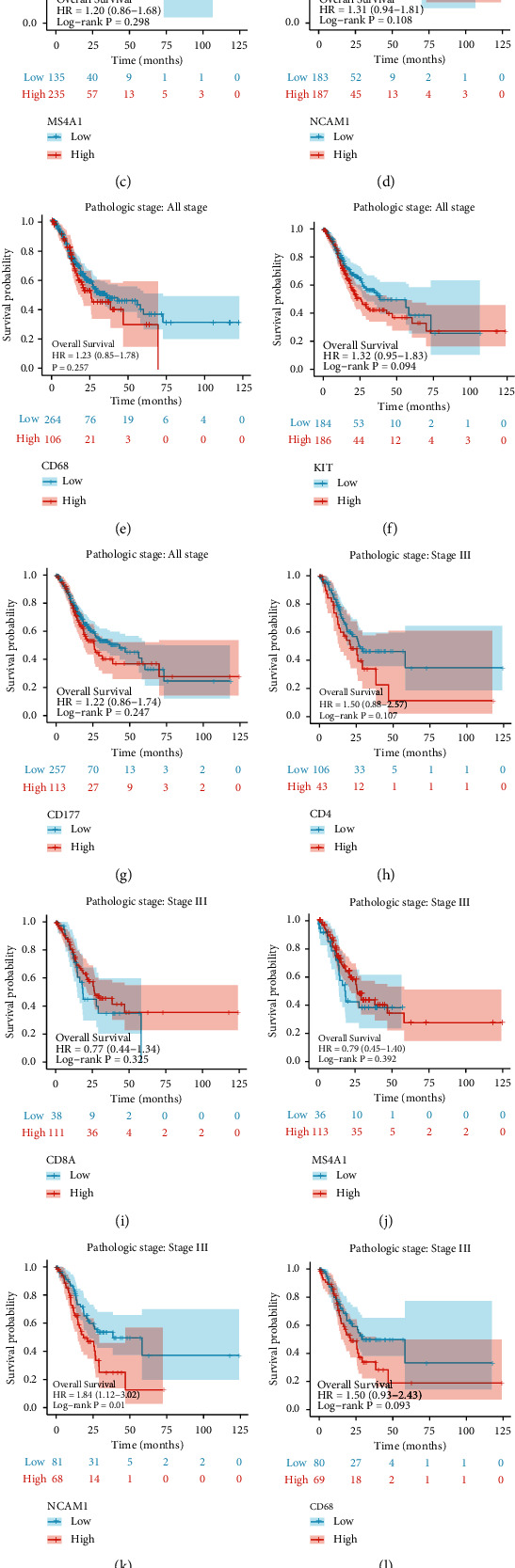
Kaplan-Meier analysis of OS probability according to the expression of immune cell surface antigen in transcriptome level from TCGA datasets. (a–g) Patients in I, II, III, and IV stages. (h–n) Patients in stage III.

Then, we analyzed the relationship between the mRNA expression level of these immune cell surface antigen and prognosis in TCGA-GC datasets, but there was no significant statistical significance (Figures 3(a)–3(g)). Considering the high tumor heterogeneity of GC, we selected the data of TCGA patients in stage III for further analysis. We found that the expressions of CD4 (Figure 3(h)), CD8 (Figure 3(i)), CD20 (Figure 3(j)), and CD68 (Figure 3(l)) were not correlated with survival probability; the expressions of CD56 (Figure 3(k)), CD117 (Figure 3(m)), and CD177 (Figure 3(n)) were negatively correlated with survival probability.

**Figure 4:**
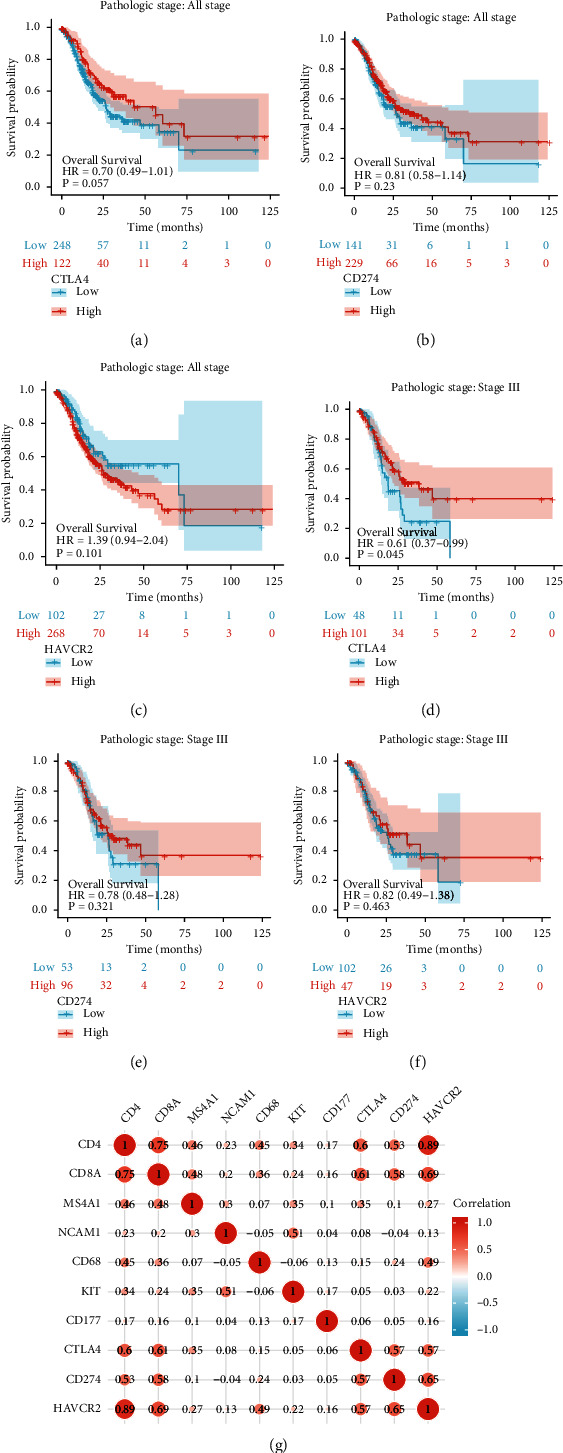
Kaplan-Meier analysis of OS probability according to the expression of immune checkpoint in transcriptome level from TCGA datasets. (a–c) Patients in I, II, III, and IV stages. (d–f) Patients in stage III. (g) Correlation between immune checkpoints and immune cell surface antigens.

Finally, we analyzed the relationship between the mRNA expression levels of immune checkpoint including CTLA-4, PD-L1 (CD274), and TIM-3 (HAVCR2) and prognosis in the TCGA-GC datasets. There was no statistically significant difference except CTLA-4 in stage III (Figure 4(a)–4(f)).  Figure 4(g) shows the correlation between immune checkpoints and immune cell surface antigens; unsurprisingly, T cells still play a crucial role in cancer immunotherapy.

### 3.3. Prognostic Value of Immune Cell Infiltration

**Figure 5:**
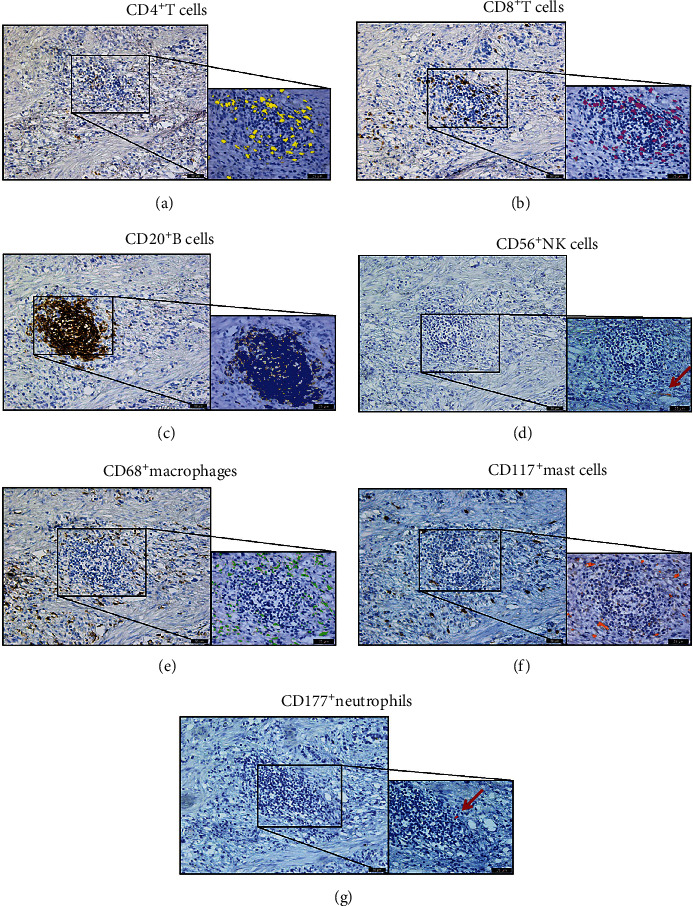
Expression of CD4, CD8, CD20, CD56, CD68, CD117, and CD177 by immune cells infiltrated tumor tissues of GC patients. Representative images of immune markers staining in immune cells from GC samples are shown at ×200 (100 mm) original magnification. Positive expression of CD4 (a), CD8 (b), CD20 (c), CD56 (d), CD68 (e), CD117 (f), and CD177 (g) infiltrated in tumor tissues with a regular distribution. In order to show the distribution from immune cells more clearly, we used computerized imaging system Image-Pro Plus version 6.2 to highlight positive areas in different colors.

**Figure 6:**
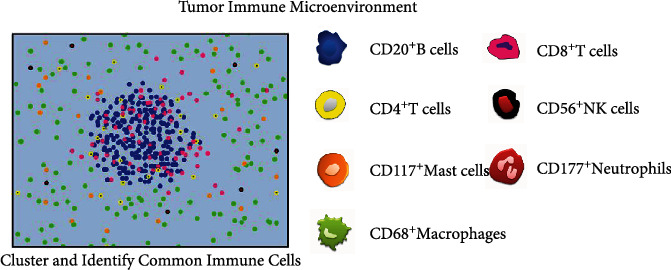
The schematic diagram of the distribution of immune cells. The blue points represent CD20^+^ B cells, the yellow points represent CD4^+^ T cells, the orange points represent CD117^+^ mast cells, the green points represent CD68^+^ macrophages, the pink points represent CD8^+^ T cells, the black points represent CD56^+^ NK cells, and the red points represent CD177^+^ neutrophils. Although not all immune cells are distributed according to this fixed law, this pattern can basically reflect the general characteristics of their distribution.

The transcription level of these immune cell surface antigens in GC patients could roughly reflect the degree of immune infiltration in the tumor microenvironment. In order to verify whether the effects of immunohistochemistry and mRNA levels on prognosis were consistent, we performed immunohistochemical staining in the tissues of patients in group A (survival time was less than 1 year) and group B (survival time was more than 5 years). All seven antibodies were specifically expressed on cell membrane. Different immune cells had different distribution characteristics in pathological section. Most CD20^+^ B cells (Figure 5(c)) were densely distributed in the form of cell clusters in central regions of lymphocytes. The CD4^+^ T cells (Figure 5(a)) scattered around B cells and then outward CD8^+^ T cells (Figure 5(b)) always distributed in strips, while CD68^+^ macrophages (Figure 5(e)) were widely distributed in the stroma of tumors. CD56^+^ NK cells (Figure 5(d)), CD117^+^ mast cells (Figure 5(f)), and CD177^+^ neutrophils (Figure 5(g)) were scattered in the stroma of tumors. This situation was represented in the schematic diagram in Figure 6.

**Figure 7:**
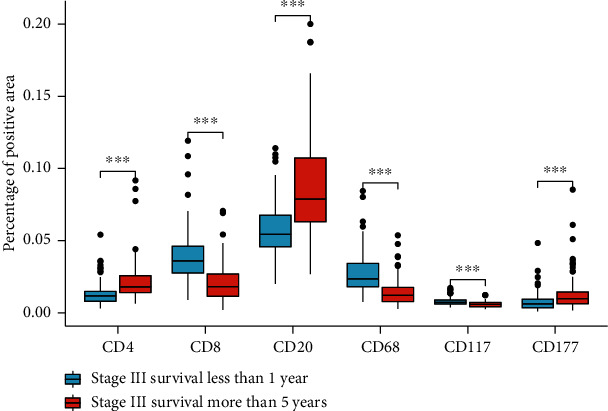
Differences in immune marker positive area/total area between the two groups (survival time of patients in group A was less than 1 year and group B survival time was more than 5 years) by the rank sum test. (a) CD4^+^ T cells (*P* < 0.001). (b) CD8^+^ T cells (*P* < 0.001). (c) CD20^+^ B cells (*P* < 0.001). (d) CD68^+^ macrophages (*P* < 0.001). (e) CD117^+^ mast cells (*P* < 0.001). (f) CD177^+^ neutrophils (*P* < 0.001).

We performed an immunoinfiltration assessment by analyzing the average positive area of the representative regions in order to reduce the difference in spatial distribution of immune cells (Figure 7). Group A was stage III patients with a survival time of less than one year, and group B was stage III patients with a survival time of more than 5 years. We found CD4+ T cells (0.95% ± 1.44% vs. 1.33% ± 0.81%), CD20^+^ B cells (5.78% ± 1.90% vs. 8.62% ± 3.47%), and CD177^+^ neutrophils (0.75% ± 0.66% vs. 1.37% ± 1.24%) infiltrated more in group B; CD8^+^ T cells (3.95% ± 1.88% vs. 2.07% ± 1.30%), CD68^+^ macrophages (1.09% ± 1.43% vs. 0.66% ± 0.92%), and CD117^+^ mast cells (0.77% ± 0.29% vs. 0.57% ± 0.21%) infiltrated more in group A. Although CD58^+^ NK cells had certain significance in predicting prognosis in the mRNA level, no statistical significance was found at the immunohistochemical level.

## 4. Discussion

GC is a highly heterogeneous cancer [26, 27]. Although most patients have the opportunity to receive radical gastrectomy in early or advanced stages, the clinical outcomes of patients from the same stage may be significantly different [28]. For example, although it was rare, some patients with advanced stage cancer can remain stable for years [29]. In contrast, more than 10% of stage I and II patients who received radical surgery died within 1 year without distant metastasis. In particular, our study showed that GC patients only in stage III had extremely different prognostic features. And it is difficult to accurately predict the outcomes of these patients after radical gastrectomy according to the present AJCC/UICC-TNM classification. Although the TNM staging system is currently the most widely accepted method, it is only based on tumor cell characteristics. However, there is growing evidence that tumor progression depends not only on tumor burden, lymph node, and metastasis but also on different distribution characteristics of immune cells [30]. As Pages et al. [31] published in Lancet in 2018, the Immunoscore provided a reliable risk assessment for recurrence in colon cancer patients, and the study supported the use of Immunoscore as a part of the new TNM-immunization classification. Therefore, tumor-associated immune cells not only reflect the tumor local state but also are effective predictors of metastasis, recurrence, and outcomes. It is necessary to proceed with in-depth study of immune cells in the stroma of GC tissues and incorporate the immune index into the GC staging system.

It is generally believed that certain immune cells in tumor tissues can secrete a large number of inflammatory factors, reactive oxygen species to destroy the matrix in the tumor microenvironment [32], kill normal cells, and promote tumor invasion [33–35]. On the other hand, some kinds of immune cells can also release immune effector molecules to target and kill tumor cells. These two-sided functions have also led to the controversy over the net influence of tumor-associated immune cell on the prognosis of GC [36–39]. The combination of multiple biomarkers in the network will significantly improve the prognostic value instead of a single biomarker. However, due to the insufficient sample size and limited conventional research methods, there is still no widely accepted Immunoscore signature system in the clinical staging of GC.

At present, limited by different incidence and geographical distribution of GC, the results of individual research centers were inconsistent, which also leads to the debate on the influence of tumor-infiltrating immune cells on GC and makes it difficult to propose a widely accepted evaluation system. On the other hand, due to the complexity of immune response, it is difficult to demonstrate the interaction between immune cells and GC cells in animal models or cell culture. Therefore, data from RNA-Seq may provide a more realistic context for researchers [40]. In this study, we attempted to explore from the level of mRNA, using publicly available gene sequencing big data.

We extracted GC mRNA expression data from the TCGA database and analyzed the relationship between immune cell surface-specific antigen and TNM stage as well as the prognoses. Coincidentally, the mRNA expression of these immune cell surface antigens was closely related to the T stage which was associated with local infiltration. It is well known that tumor progression relies on a dense network of interactions between cancer cells and the surrounding stroma, niche-defining cells, and vasculature. One of the most important aspects of tumor-microenvironment crosstalk is the ability of cancer cells to modulate inflammatory responses through soluble mediators. This may allow us to see an increase in immune cell infiltration as tumor stage progresses. This analysis was based on 375 patients with TCGA mRNA data which had been processed by a unified standard and makes the result more reliable. Then, we analyzed the correlation between the expression of immune cell surface antigen and survival time in stage III GC. It was found that the high expression of CD56, CD117, and CD177 predicted poor prognosis by the Kaplan-Meier analysis. The effects of cancer cell activity on the innate and adaptive immune systems are multifaceted. Recent studies [41] have pointed to abnormal growth and activity of the microbiota as a cause of increased inflammation and consequent tumor growth. The development of dysbiosis of the tumor microenvironment appears to be a major contributor to inflammation-related tumor growth, in which dysregulation and enhanced microbial translocation of the gastric mucosal barrier are secondary to chronic inflammation or tumors and promote cancer development.

To be more clinically relevant, we validated the result from the level of immunohistochemistry. In consideration, immunohistochemistry has inherent complexity, such as the difference of organizational selection criteria, experimental conditions, and quantitative criteria, which might contribute to the variability of the results obtained. A single-center study could avoid many heterogeneous factors if robust data can be obtained. Before our research, Jiang et al. [42] reported that the Immunoscore is expected to be a prognostic and predictive tool in GC. They used the LASSO model and established an IS GC classifier based on 5 features: CD3 invasive margin (IM), CD3 center of tumor (CT), CD8 IM, CD45RO CT, and CD66b IM. Wen et al. [24] pointed out that the immune indicators can guide the sensitivity of postoperative chemotherapy. However, most of the current studies do not consider the impact of tumor staging on the immune microenvironment. The tumor immune microenvironment may change with different stages of the tumor [43, 44], and this change often leads to different experimental results, which also leads to the discussion of the impact of current immune indicators on GC patients [45, 46]. In 2018, *CELL* [47] described for the first time in breast cancer that the distribution of immune cells and tumor cells exhibited three characteristics: no infiltration at all, mixed infiltration, and intermittent infiltration. Their study also found that the prognosis of patients with intermittent infiltration was significantly higher than that of mixed infiltration. Although our study only focused on immune cells, we found that the spatial infiltration regularity of these immune cells was roughly as described in our results. B cells in the gastric cancer stroma always seem to be at some distance from the tumor cells and exist in clusters. However, macrophages and neutrophils appear to be more closely related to tumor cells.

Our initial study through extensive follow-up data found that the prognosis of stage I and II GC is basically consistent with the prediction of current TNM stage, but the prognosis of patients with stage III GC often has an extreme difference. This may be because stage III GC is more susceptible to the immune microenvironment than early stage. Therefore, studies focusing on the immunology of GC with stage III may be more likely to be widely accepted. In order to minimize the randomness of pathological materials and the subjective feature in immunohistochemical analysis, we randomly selected two groups of patients with severe prognosis of stage III GC in a population with large sample size while strictly controlled the materials and experimental conditions, to screen for immune cells that could represent different prognosis. Considering the applicability and convenience, an ideal marker should be feasible, inexpensive, repeatable, quantifiable, standardizable, and uncomplicated.

In our study, T helper cells were marked by CD4, cytotoxic T cell by CD8, B cells by CD20, NK cells by CD56, macrophages by CD68, mast cells by CD117, and neutrophils by CD177. We found that these immune cells were all associated with poor prognosis in addition to CD20^+^ B cells, CD4^+^ T cells, and CD177^+^ neutrophil (there was no correlation between the infiltration of CD56^+^ NK cells and prognosis). Although the number of patients with stage IIIA GC in group A was more than that in group B, and the number of patients with stage IIIC GC in group A was less than that in group B according to the current TNM stage, it was not enough to explain the significant difference in prognosis of stage III. Our research confirmed that the degree of infiltration of immune cells in the tumor microenvironment is meaningful in evaluating the patients with stage III GC. Considering that patients with stage III GC receive chemotherapy after surgery, the assessment of immune infiltration is expected to distinguish those patients who are not sensitive to chemotherapy, which can help them avoid the overtreatment and the side effects of chemotherapy drugs. The Cancer Genome Atlas Research Network divided GC into four subtypes: Epstein-Barr virus- (EBV-) positive, microsatellite instability, genomically stable, and chromosomal instability [48]. There was a significant difference in immune cell infiltration in different GC molecular types determined by pathologists. For example, Epstein-Barr virus-positive and microsatellite unstable GC cases had more lymphocytic infiltration [49]. Therefore, future studies should focus on the association between immune infiltration and molecular classification.

With the development of gene sequencing, it is widely accepted that the data from transcriptome level could bring us closer to cancer cells without considering their clinical progression [50–52]. Although traditional pathological immunohistochemistry has been used for hundreds of years, its clinical application is still unmatched by any other kind of detection technology [53, 54]. In this study, we explored the significance of immune cells in the prognosis of GC tissue and further incorporated sophisticated gene sequencing technology and traditional immunohistochemical techniques. Both grading systems had certain significance for predicting the prognosis of patients with GC. We found that different infiltration patterns of immune cells can predict the clinical prognosis of patients with GC in stage III. At the level of immunohistochemistry, CD8, CD68, and CD117 expressions were associated with poor prognosis; CD4, CD20, and CD177 expressions were associated with better prognosis. It pointed that CD117^+^ mast cells have the same trend of predicting significance for prognosis at the RNA and protein levels by analyzing the correlation between the two levels of various types of immune cell surface antigen and prognosis. The mysterious role of mast cells in cancer has long been debated. The role of mast cells in promoting or restricting tumor growth often varies according to the tumor microenvironment [55]. When exerting an anticancer effect, mast cells release a variety of inflammatory mediators that help resolve infection and fight inflammation but also have potency in promoting or inhibiting malignancy. In view of this remarkable plasticity of mast cells, further research was necessary. For CD177, the trend of prognostic significance was opposite at RNA level and protein level. This may be due to the fact that the mRNA abundance of some specific genes may not be linearly related to the protein expression of its translation products. A multicenter study [56] published in the *Annals of Surgery* evaluated the relationship between neutrophil infiltration and clinical outcomes in gastric cancer patients receiving adjuvant chemotherapy. The results showed that patients with high neutrophil infiltration were easier to have longer OS compared with those with low infiltration, suggesting that this may be an important predictor of chemotherapy efficacy. There are many regulatory levels of gene expression. Posttranscriptional regulation and translation play important roles in the final protein expression. Wennemers et al.'s [57] study found that there were significant differences between mRNA level and protein expression, which could be related to the regulation of protein degradation rate better than mRNA. In addition, the degradation of mRNA and the degradation, modification, and folding of protein may lead to the inconsistency between mRNA abundance and protein expression.

As a retrospective study, there were several limitations in our study. First, this study only focused on an Asian population in a single center. Whether the results are widely applicable to white and black populations needs to be further studied by expanding the sample size. Secondly, our research study has not yet established an effective prediction model and only proposed which kind of immune cells are more suitable for the evaluation of stage III GC, and large-scale data is necessary for the validation. It is difficult to unify the standard of resection. Although we have made detailed records for each operation, this technical problem is inevitable.

## 5. Conclusions

In conclusion, our study had for the first time pointed out that patients with GC in northeastern China have significant prognostic differences only in stage III. This required us to find a more effective way to predict survival for these patients. Although our results were different from those of previous studies, due to the high heterogeneity of GC, future assessments of GC Immunoscore should take into account different regions and cancer stages. The CD117^+^ mast cells may be important evaluation factors in Immunoscore.

## Figures and Tables

**Table 1:** Patient characteristics.

Characteristics	Group A	Group B	*P* value
Sex			0.033
Male	82	69	
Female	18	31	
Age^a^			0.161
	60.33 ± 11.44	58.12 ± 10.75	
CA19-9^a^			<0.001
	125.73 ± 242.39	24.17 ± 73.37	
CEA^a^			0.083
	19.79 ± 78.18	5.96 ± 14.07	
Albumin^a^			0.191
	38.92 ± 5.00	39.77 ± 4.13	
Neutrophil-lymphocyte percentage^a^			0.044
	2.88 ± 2.40	2.31 ± 1.48	
Borrmann type			<0.001
I	0	5	
II	13	34	
III	61	52	
IV	26	9	
AJCC stage^b^			<0.001
IIIA	19	51	
IIIB	34	37	
IIIC	47	12	

^a^Values are mean ± standard deviation. ^b^Tumor staging according to the 8^th^ American Joint Committee on Cancer/International Union Against Cancer classification.

## Data Availability

The datasets used in this study are available from the corresponding author on reasonable request. More information can also be obtained from the Gastric Cancer Information Management System v1.2 of the Harbin Medical University Cancer Hospital (Copyright No. 2013SR087424, http://www.sgihmu.com/).
